# Domain-guided data augmentation for deep learning on medical imaging

**DOI:** 10.1371/journal.pone.0282532

**Published:** 2023-03-23

**Authors:** Chinmayee Athalye, Rima Arnaout

**Affiliations:** 1 Division of Cardiology, Department of Medicine, Bakar Computational Health Sciences Institute, University of California San Francisco, San Francisco, California, United States of America; 2 Division of Cardiology, Department of Medicine, Department of Radiology, Bakar Computational Health Sciences Institute, Computational Precision Health Graduate Program, Center for Intelligent Imaging, Biological and Medical Informatics Graduate Program, Chan Zuckerberg Biohub Intercampus Research Award Investigator, University of California San Francisco, San Francisco, California, United States of America; Vellore Institute of Technology: VIT University, INDIA

## Abstract

While domain-specific data augmentation can be useful in training neural networks for medical imaging tasks, such techniques have not been widely used to date. Our objective was to test whether domain-specific data augmentation is useful for medical imaging using a well-benchmarked task: view classification on fetal ultrasound FETAL-125 and OB-125 datasets. We found that using a context-preserving cut-paste strategy, we could create valid training data as measured by performance of the resulting trained model on the benchmark test dataset. When used in an online fashion, models trained on this hybrid data performed similarly to those trained using traditional data augmentation (FETAL-125 F-score 85.33 ± 0.24 vs 86.89 ± 0.60, p-value 0.014; OB-125 F-score 74.60 ± 0.11 vs 72.43 ± 0.62, p-value 0.004). Furthermore, the ability to perform augmentations during training time, as well as the ability to apply chosen augmentations equally across data classes, are important considerations in designing a bespoke data augmentation. Finally, we provide open-source code to facilitate running bespoke data augmentations in an online fashion. Taken together, this work expands the ability to design and apply domain-guided data augmentations for medical imaging tasks.

## Introduction

First demonstrated for non-medical tasks, deep learning has shown remarkable utility for medical imaging in recent years [1–3]–with little to no need to adapt neural network architectures for medical domains. The use of augmentation techniques to enhance the diversity of available training data critical to training robust and generalizable deep learning models [4]. Traditional data augmentations for images include shear, rotation, flipping, blurring, contrast stretching, and other operations [5] performed in an online manner during training (i.e., computing slightly different data augmentations on each image on-the-fly during training, so that each time the image is used, it looks slightly different).

While neural network architectures can be applied off-the-shelf to medical imaging tasks, the same is not always true for data augmentation techniques for two reasons. First, domain expertise is needed to apply traditional data augmentations correctly. For example, flipping X-rays can change the laterality of the image; rotating images significantly can change the training labels in adult echocardiography [6] but can be very helpful in fetal ultrasound [1]; and a high degree of distortion can affect image quality in ways that are not clinically relevant or can obscure important anatomic structures. The spectrum of traditional augmentations/hyperparameters that can be used in a given medical imaging task is therefore constrained, and model training can face data starvation.

Second, traditional data augmentations do not fully exploit the domain-specific traits of medical images. This means that opportunities to further expand the training dataset may not be leveraged. Furthermore, failing to apply domain-specific data augmentation can lead to fundamental bias in trained models, such as relying on fiducial markings associated with skin biopsies predict skin cancer [7], or relying on presence of endotracheal tubes or other equipment to predict severity of disease from chest X-rays [8]. To fully exploit domain-specific traits, domain-guided data augmentation can be useful for medical imaging. However, it has not been widely used to date due to complexity of implementation compared to traditional methods.

Our objectives were to test whether domain-guided data augmentation can be used to successfully train deep learning tasks in medical imaging, to compare performance of domain-guided vs. traditional data augmentation in benchmarked medical imaging tasks, and to highlight important design and implementation considerations when using domain-guided data augmentation. We present a custom, context-preserving, anatomy-aware way of combining two images in an online fashion to create new training data for medical imaging.

To illustrate the development and evaluation of domain-guided data augmentation, we used a well-benchmarked [1] task of fetal cardiac view classification, where five screening views of the fetal heart—called 3-vessel trachea (3VT), 3-vessel view (3VV), left-ventricular outflow tract (LVOT), axial 4-chamber (A4C), or Abdomen (ABDO)—must be distinguished from non-target (NT) images. Both class imbalance (see Methods) and the laborious nature of labeling motivate a desire to augment the training dataset.

The defining features of each screening heart view are found within the axial thorax, while features outside the thorax (e.g. arms, legs, umbilical cord) are non-specific. We therefore chose cutting-and-pasting thoraces from one image into another as our domain-guided data augmentation strategy to create hybrid images. Cut-paste for a deep learning application was first used for instance detection [9] and has been shown to improve performance [9–12]; some suggest that random positioning [9, 12] of pasted objects performs well, while others have shown that context-aware approaches [10] are important. CutMix [13] and Mixup [14] are cut-paste approaches that have been used in non-medical settings, but neither creates visually realistic images. For medical imaging, cut-paste data augmentation has been used in an offline manner to generate instances with lesions in chest CT scans [15]. However, this method used a complex blending tool based on Poisson image editing which cannot be scripted to generate images in an online manner. This is a disadvantage, because the ability to perform data augmentation in an online manner provides regularization which is critical to training neural networks. TumorCP used an online cut-paste strategy for the task of kidney tumor segmentation [16]. However, the test set was small (only 42 images) and performance variable (up to 28 percent standard deviation in reported Dice score). Similar to TumorCP, the fetal task involves cutting the thorax from one image and pasting it into another and preserves anatomical context so that new images do not require relabeling by clinicians. In contrast, the fetal task is multi-class and has a much larger test set.

We used the fetal multi-classification use case to test whether domain-specific data augmentation was useful for deep learning tasks in medical imaging. We report on design and performance, compared to traditional data augmentation.

## Methods

### Dataset

The training data consists of still frames from fetal screening ultrasounds and fetal echocardiograms, as in [1]. This dataset is imbalanced across the six classes– 3193 (6.1 percent of total training data) 3VT, 6178 (11.8 percent) 3VV, 6735 (12.9 percent) LVOT, 6029 (11.5 percent) A4C, 5206 (9.9 percent) ABDO, and 25082 (47.8 percent) NT images. Data were accessed under UCSF IRB 17–21481 with waived consent.

Two test datasets were used. The FETAL-125 test set consists of images from 125 fetal echocardiograms: dedicated ultrasounds of the fetal heart, performed by fetal cardiologists and sonographers with specific expertise in fetal cardiology. The OB-125 test set consists of fetal screening ultrasounds (second-trimester obstetric anatomy scans performed by sonographers, radiologists, obstetricians and/or maternal–fetal-medicine physicians) corresponding to the same patients as in FETAL-125. The FETAL-125 test set [1] consists of 306 (2.7 percent of this test set) 3VT, 890 (7.8 percent) 3VV, 1800 (15.7 percent) LVOT, 3521 (30.8 percent) A4C, 563 (4.9 percent) ABDO, and 4365 (38.1 percent) NT images for a total of 11,445 images. The original OB-125 test set [1] consists of 678 (0.3 percent) 3VT, 2431 (1.1 percent) 3VV, 3755 (1.7 percent) LVOT, 16852 (7.6 percent) A4C, 3473 (1.6 percent) ABDO, and 193801 (87.7 percent) NT images—this is because the fetal screening ultrasound contains many more images of non-cardiac structures. This vast class imbalance in OB-125 makes it hard to use any changes in the F-score or accuracy for comparing performance between experiments. Hence, we randomly sampled a subset of the OB-125 that had 678 images for each target view class and 3390 NT images; this ‘subsampled OB-125’ test set was used and henceforth will simply be called ‘OB-125.’

### Hybrid image generation

**Fig 1:**
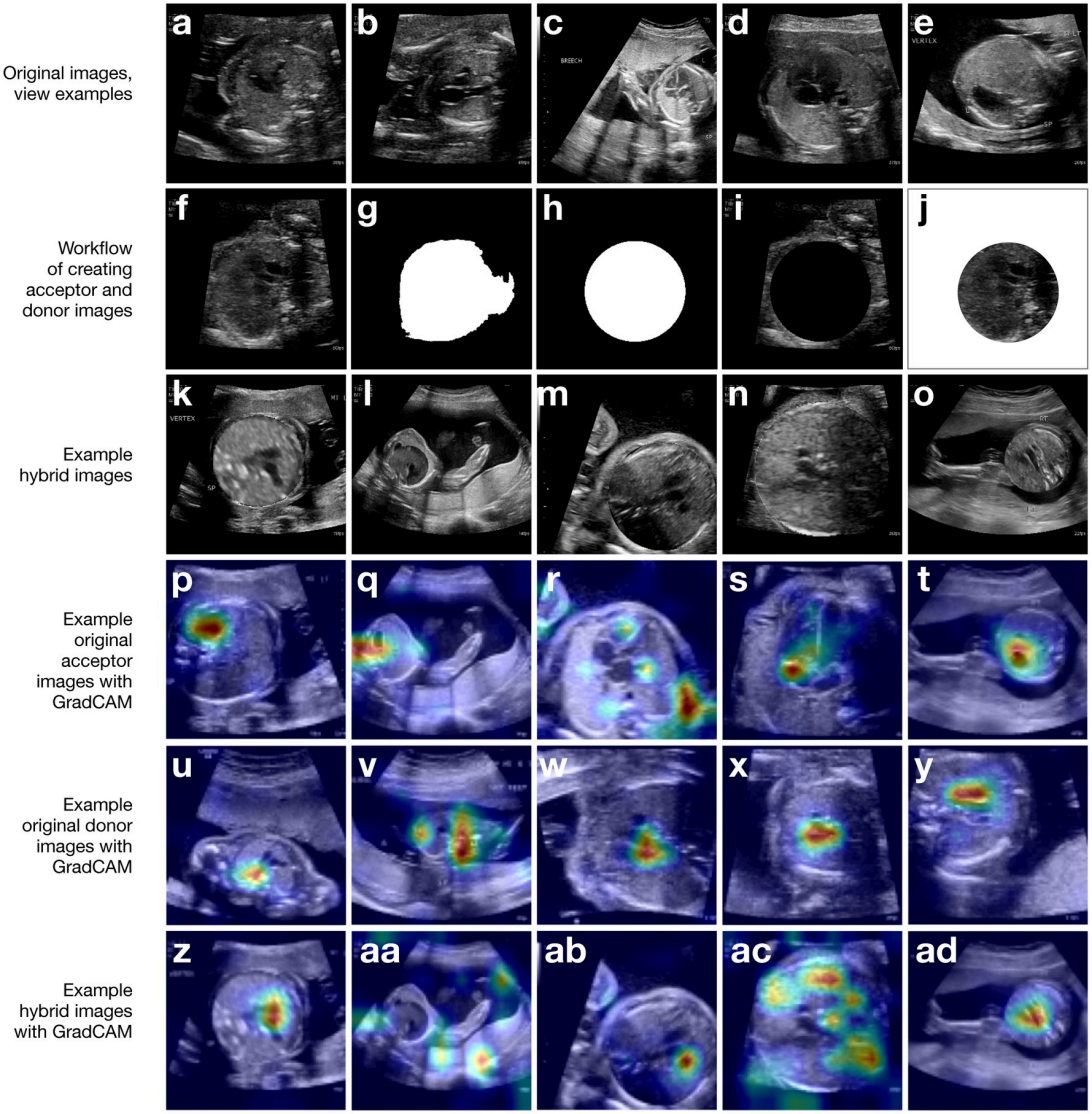
Image examples and workflow for applying a bespoke, cut-paste data augmentation to images. **(a-e)** show examples of the three-vessel trachea (3VT), three-vessel view (3VV), left ventricular outflow tract (LVOT), axial four-chamber (A4C), and abdomen (ABDO) views, respectively. **(f-j)** demonstrate the workflow of using an original training image **(f)** to detect the thorax **(g)** and perform quality control to create a mask **(h)** that can be used to generate an acceptor **(i)** and a donor **(j)**. **(k-o)** show five examples of hybrid images. **(p-t)** show the original acceptor images for these hybrids, overlaid with gradient-weighted class activation maps (GradCAMs) from model inference. **(u-y)** show the original donor images for these hybrids, overlaid with GradCAM. **(z-ad)** show the GradCAM for the hybrid image examples.

The workflow to generate a cut-pasted hybrid image is described in Fig 1a. We used a thorax detector segmentation model described in [1] to extract the region of interest in each image. We used the convex hull of the segmented thorax region, closing any holes in the segmented region and removing any extraneous pixels. To make the extraction and combination appear seamless, we approximated the thorax to its best fit circular region (an axial thorax should be round), rejecting highly eccentric (> 0.75 eccentricity) segmentations. We define the eccentricity as the ratio of the major axis to minor axis of the convex hull. We then used a binary mask to separate the image into two components–the thorax (donor) and the background image with a cavity (acceptor) in place of the thorax as shown in Fig 1a. All images from which we can extract a valid donor and acceptor are deemed cut-paste eligible. Hybrid images were then created by randomly choosing a donor and an acceptor, resizing the donor thorax to the size of the acceptor cavity, randomly rotating the thorax 10–350 degrees, and pasting the donor region in the acceptor cavity. The new hybrid image carried the same class label as the donor thorax. All these steps were performed using Python 3.6, with scikit-image version 0.16.2 and OpenCV version 4.5.1 packages.

### Training parameters

For all experiments, the following architecture and training parameters were used. These training parameters were also the same for the original model in [1]. We used the ResNet architecture [17] for the view classifier with an input image size of 80 by 80 pixels. The batch size was set to 32. We used the Adam optimizer with a starting learning rate of 0.0005 and then adaptively reduced the learning rate by a factor of 0.5 if there was no improvement greater than 0.001 in the validation loss for 5 consecutive epochs. The maximum number of epochs was set to 250 with early stopping if there was no improvement greater than 0.001 in the validation loss for 15 consecutive epochs. Dropout of 50 percent was applied before the final fully connected layer. All models were implemented in Python 3.6 and TensorFlow 2.1.3 Keras 2.3.0 framework. We executed the training on an AWS EC2 ‘g4dn.4xlarge’ instance with a Nvidia T4 GPU and 64GB memory.

### Traditional data augmentation

For all experiments where traditional data augmentation was used, online data augmentation consisted of applying Gaussian blur and rescaling the image intensity to within 2^nd^ and 98^th^ percentile with 50 percent probability each, rotating images for up to 10 degrees, width and height shift of up to 20 percent of original size, zooms of up to 50 percent, and vertical and horizontal flips at random. Traditional data augmentation was the same as in the original model [1].

### Original model

As in [1], the original model made use of all original training data above, the neural network and training parameters above, and the traditional data augmentation described.

### Statistical testing

For statistical rigor, three replicates were performed for each experiment [18–20]. We used two-tailed t-tests to compare performance between experiments. A one-tailed t-test was used to compare an experiment with the benchmark in [1].

## Results

To test whether a domain-guided data augmentation approach could produce valid training data for deep learning applications, we performed several experiments using the thorax cut-paste augmentation strategy on fetal ultrasound images as a use case, evaluating using the benchmark task of six-view classification with ResNet. We evaluated the models on two test sets: (1) fetal echocardiograms from 125 patients formed the FETAL-125 test set and (2) a class-balanced subset (see Methods) of the corresponding patients’ fetal screening ultrasounds formed the OB-125 test set.

As an overview, we first generated hybrid images offline (before model training). We then tested whether they could be used to train a view classifier without overfitting and with reasonable performance. In this case, traditional data augmentation was applied during model training to provide regularization. We then implemented hybrid image generation online during model training, so that hybrid image generation could supply both data augmentation and training-time regularization. We tested whether online hybrid image generation was sufficient to provide regularization. We then compared performance of a model trained with online hybrid generation, to a model trained with traditional online data augmentation as a control.

### Cut-paste technique produces images that are valid and are useful in training

We first tested whether our hybrid image generation method could produce valid training data by synthesizing images offline, and then using them to re-train our benchmark classification task. Using our pipeline (Methods, Fig 1a), we created new hybrid images that were realistic overall with only small cut-paste combination artifacts (Fig 1b). Adjacent frames in an ultrasound video from the same patient will generally have very similar heart structure [21], so we randomly chose only one frame per view per patient ID for target views and five frames per patient ID for NT view to generate a set of candidate images; these frames also had to pass quality control (Methods). Thus, we used *only five percent* of the original training data to synthesize thousands of new images such that the new hybrid training dataset was approximately the size of the original training dataset (Table 1).

**Table 1:** Distribution of training data with 90 percent hybrid images.

View	1. Number of original training images	2. Number (percent) of donors	3. Number of times each donor is sampled	4. Number of hybrid images generated	5. Number (percent) of original training images sampled	6. Total number of training images	7. Percent of hybrid images in total training data
3VT	3193	297 (9.3)	22	6534	700 (22)	7234	90.3
3VV	6178	439 (7.1)	15	6585	700 (11)	7285	90.4
LVOT	6735	469 (7.0)	14	6566	700 (10)	7266	90.4
A4C	6029	570 (9.5)	12	6840	700 (12)	7540	90.7
ABDO	5206	475 (9.1)	14	6650	700 (13)	7350	90.5
NT	25082	633 (2.5)	50	31650	3500 (14)	35150	90.0
TOTAL	52423	2883 (5.5)	N/A	64825	7000 (13)	71825	90.2

Distribution of training data where 90 percent of training data consists of hybrid images generated in an offline manner (columns 6, 7). Only about five percent (column 2) of the original training images (column 1) are used to generate all the new hybrid training images (column 4). Overall, the entire hybrid training dataset (column 6) was derived from only 13 percent of the original images. Column 3 denotes the number of times each donor is samples. A total number is not applicable here.

To prevent the model from overfitting on any combination artifacts, all view classes had an approximately equal percentage of hybrid images: about 90 percent. This meant that the classes with lower numbers of donors were sampled more times than others (Table 1). The remaining 10 percent of images were original (non-hybrid). Overall, the entire hybrid training dataset was derived from only 13 percent of the original images (Table 1).

We re-trained our model using the hybrid training dataset described above and the hyperparameters and traditional data augmentation as in the Methods. The model trained on the new data set without overfitting. Table 2 shows that accuracy and F-score for the models trained on this hybrid dataset is comparable with the original model (FETAL-125 F-score and accuracy p-values both 0.45; OB-125 F-score and accuracy p-values both 0.12). As with the original dataset [1], this hybrid-trained model performs slightly better on FETAL-125 (higher-quality fetal echocardiogram images) compared to OB-125 (screening ultrasound images) (F-score 91.99 ± 2.62 vs 80.76 ± 0.99, accuracy 97.33 ± 0.87 vs 93.58 ± 0.33; both p-values 0.002). By recapitulating original model performance using a dataset overwhelmingly composed of hybrid images, we demonstrate that the cut-paste strategy is a viable method to generate additional training images.

**Table 2:** Performance of model trained on 90 percent hybrid data generated offline.

Metric	FETAL-125			OB-125		
	Original model	90 percent hybrid training data (mean±SD)	p-value	Original model	90 percent hybrid training data (mean±SD)	p-value
Accuracy	**97.80**	97.33 ± 0.87	0.45	93.09	**93.58 ± 0.33**	0.12
F-score	**93.40**	91.99 ± 2.62	0.45	79.27	**80.76 ± 0.99**	0.12

Original model was benchmarked in [1]. p-values reported using the two-tailed t-test. Hybrid images were generated offline, and traditional data augmentation was applied at training time for regularization.

### When used in an online manner, the cut-paste technique provides the regularization needed to train a neural network

Online data augmentation acts as a regularization technique while training a neural network [4]. In the previous experiment, the hybrid images were shown to serve as valid training data, while regularization was provided from online traditional data augmentation. We next tested whether, when performed in an online manner, hybrid images could also provide the necessary regularization to avoid overfitting (without need for traditional data augmentation). We used all cut-paste eligible images from the training data, or 80.1 percent of the original training images (Table 3). Implementing the cut-paste strategy in an online manner generates a new set of hybrid images per epoch due to the random combinations. With our cut-paste eligible data (Table 3, total number of eligible donors), it is possible to generate 1.2e9 new unique hybrid images.

**Table 3:** Number and class distribution of cut-paste eligible images.

View	Number of original training images	Number of eligible donors and acceptors	Percent of original data with eligible donors
3VT	3192	2721	85.24
3VV	6178	5482	88.73
LVOT	6735	5770	85.67
A4C	6029	5612	93.08
ABDO	5970	5206	87.20
NT	25082	10239	40.82
TOTAL	52423	35030	66.82

All hybrid eligible images contribute both a donor thorax and an acceptor cavity.

**Fig 2:**
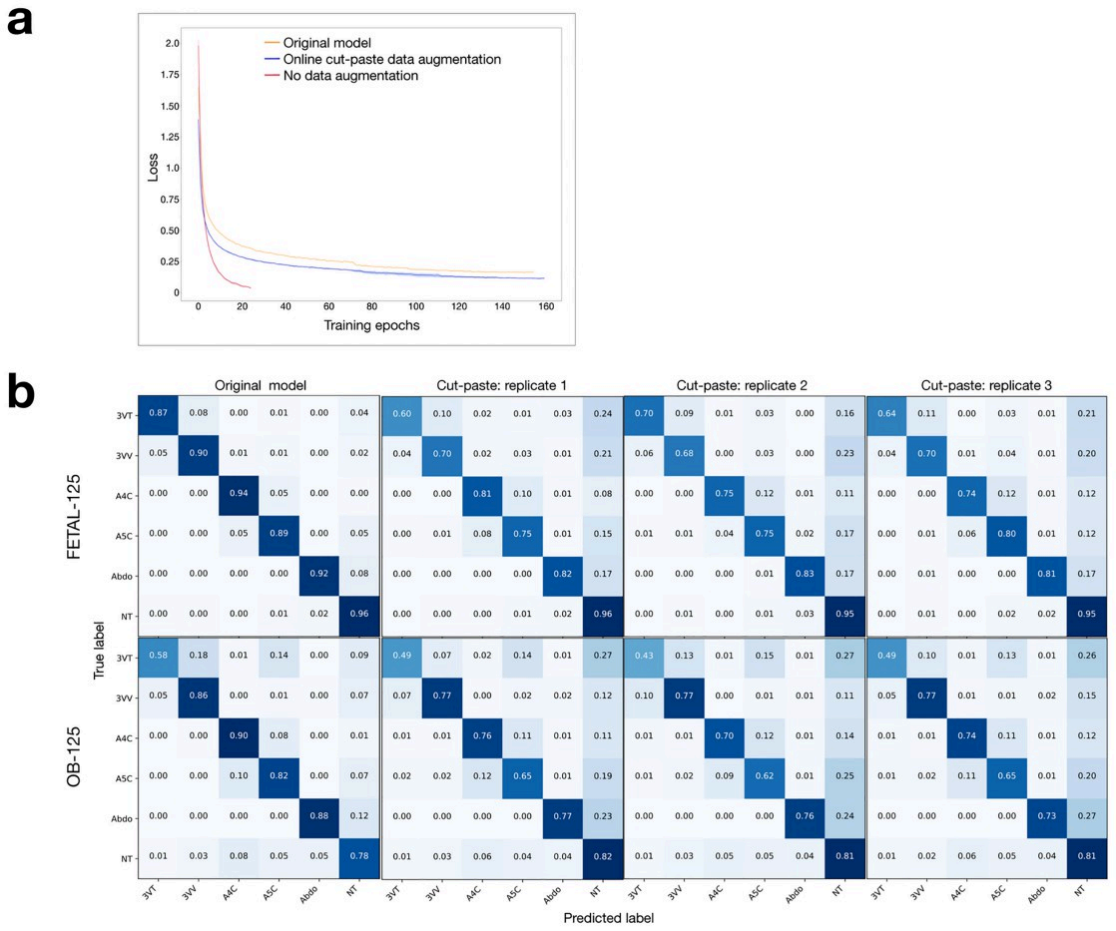
Cut-paste as a data augmentation strategy. **(a)** Loss plots for original training (yellow), training with no online data augmentation (red, with error as light red), and training with online cut-paste data augmentation (blue, with error as light blue). **(b)** Normalized confusion matrices of FETAL-125 and OB-125 test data, for original model and model trained with online cut-paste data augmentation (three replicates shown).

Fig 2a compares the training loss progress of the model when the cut-paste strategy is used as an online data augmentation, compared to the training of the original model from [1]. The training loss when no data augmentation is used is shown as a negative control, showing that the model quickly overfits the training data when no data augmentation is used. Comparison of the training loss plots show that the cut-paste strategy works better (p-value < 0.001) as an online data augmentation strategy (Fig 2). Table 4 shows the F-score and accuracy values of this newly trained model.

**Table 4:** Performance of model trained on hybrid data generated online.

Metric	FETAL-125			OB-125		
	Original model	Online cut-paste (mean±SD)	p-value	Original model	Online cut-paste (mean±SD)	p-value
Accuracy	**97.8**	94.4 ± 0.28	0.002	93.09	**91.5 ± 0.27**	0.009
F-score	**93.4**	83.1 ± 0.83	0.002	79.27	**74.4 ± 0.82**	0.009

### The ability to apply bespoke data augmentation equally across classes is an important design consideration

While the above experiment showed better training loss using the cut-paste data augmentation strategy, the confusion matrices in Fig 2b demonstrate a skew toward prediction of the NT class. We tested whether this was due to an imbalance in how the chosen bespoke data augmentation can be applied to images of different classes; only 41 percent of NT images were cut-paste eligible compared to 85–93 percent for the other view classes (Table 3), because the NT class includes many images where no thorax appears.

With cut-paste strategy implemented as an online data augmentation with probability 1 (i.e., every image eligible to undergo cut-paste processing would receive it), each batch of training data would contain up to 15 percent of target-class images in their original form with the rest as hybrid images. Due to the imbalance in eligible images in the NT class, the model would see 59 percent of the NT-class images in their original form during training. The test images are all original unchanged images.

We therefore mitigate this imbalance of hybrid and unchanged training data using a sampling strategy in the subsequent experiments to include similar proportions of online data-augmented and non-data-augmented images in each training batch, regardless of whether traditional or bespoke data augmentation is used.

### Cut-paste data augmentation performs similarly to traditional data augmentation in a classification task but produces fewer false-positive view predictions

To counter the class imbalance in application of the cut-paste technique while preserving online data augmentation, we employed a new sampling strategy for image batches during online training. All the target class cut-paste eligible images (85–93 percent) are passed as both hybrid images and in their original unchanged form to the model at every epoch. For the NT class, the cut-paste eligible images (41 percent) are passed only in the hybrid form and the rest (59 percent) in their original, unchanged form.

Similarly, for training using traditional data augmentation, images were sampled such that about 50 percent of images per training batch underwent traditional data augmentation, while the rest of the images in the batch remained unchanged. Therefore, for each view class, target classes and NT class alike, each training batch contained approximately 50 percent augmented and 50 percent original images, regardless of whether traditional or bespoke data augmentation was applied. The sampling strategy is demonstrated for a toy batch of data in S1 Table.

The overall and per-class test performance for these experiments are given in Tables 5 and 6. The new sampling strategy shows improvement in performance over the previous experiment (Fig 2) as is seen in the improved F-score and accuracy over both test sets in Table 5 (all p-values < 0.01). On the FETAL-125 test set, absolute performance from traditional and bespoke data augmentation methods is quite similar (e.g. accuracy of 95.63 ± 0.20 with traditional data augmentation vs. 95.11 ± 0.08 with bespoke data augmentation), despite statistical significance weighing in favor of traditional augmentation. On the OB-125 test set, both absolute performance and statistical significance favor bespoke data augmentation (F-score p-value 0.004, accuracy p-value 0.002).

**Table 5:** Comparison of models trained with traditional and cut-paste data augmentation when application of augmentation during training time is balanced.

Metric	FETAL-125			OB-125		
	Traditional DA (mean±SD)	Cut-paste DA (mean±SD)	p-value	Traditional DA (mean±SD)	Cut-paste DA (mean±SD)	p-value
Accuracy	**95.63 ± 0.20**	95.11 ± 0.08	0.014	90.59 ± 0.21	**91.53 ± 0.04**	0.002
F-score	**86.89 ± 0.60**	85.33 ± 0.24	0.014	72.43 ± 0.62	**74.60 ± 0.11**	0.004

Bold type indicates the higher-performing experiment. p-value reported using the two-tailed t-test. DA, data augmentation.

**Table 6:** Per-class recall values for results in Table 5.

	FETAL-125					
	Traditional data augmentation			Domain-guided data augmentation		
View	precision (mean±SD)	recall (mean±SD)	F1 (mean±SD)	precision (mean±SD)	recall (mean±SD)	F1 (mean±SD)
3VT	0.62±0.06	0.69±0.02	0.65±0.03	**0.73±0.04**	0.64±0.02	**0.69±0.03**
3VV	**0.91±0.02**	0.72±0.03	0.80±0.01	0.86±0.01	0.75±0.02	0.80±0.01
LVOT	0.80±0.03	0.77±0.02	0.79±0.01	0.78±0.02	0.77±0.05	0.77±0.02
A4C	0.95±0.01	0.86±0.03	0.90±0.01	0.94±0.01	0.83±0.02	0.88±0.01
ABDO	0.75±0.003	0.84±0.02	0.79±0.01	0.72±0.03	0.86±0.03	0.78±0.03
	OB-125					
	Traditional data augmentation			Domain-guided data augmentation		
View	precision (mean±SD)	recall (mean±SD)	F1 (mean±SD)	precision (mean±SD)	recall (mean±SD)	F1 (mean±SD)
3VT	0.65±0.06	0.46±0.03	0.53±0.02	0.74±0.02	0.49±0.01	0.59±0.01
3VV	0.72±0.01	0.69±0.03	0.71±0.02	0.72±0.003	0.80±0.03	0.76±0.01
LVOT	0.60±0.01	**0.66±0.01**	0.63±0.003	0.58±0.02	0.62±0.004	0.50±0.01
A4C	0.58±0.02	0.79±0.02	0.67±0.02	**0.63±0.02**	0.81±0.02	0.70±0.01
ABDO	0.66±0.03	0.80±0.02	0.72±0.01	**0.75±0.02**	0.79±0.04	**0.77±0.02**

Bold type indicates the higher-performing experiment with p-value <0.05; grey shading indicates the higher-performing experiment with 0.05 < p-value < 0.1.

The training loss plots for both these experiments and their replicates are shown in S1 Fig, showing that the model trained with traditional data augmentation overfit the training data which consists of majority fetal echocardiogram images. This result is consistent with the model’s better performance on the FETAL-125 test set, which consists of all fetal echo images, compared to OB-125. Additionally, the changes in per-class recall values for both these experiments are reported in Table 6. We note that the model trained with cut-paste data augmentation outperforms the model trained with traditional data augmentation on the 3VT view (FETAL-125 recall p-value 0.22; OB-125 recall p-value 0.057), the worst-performing view in the original model.

Finally, we examined the number of false-positive views in the OB-125 test set predicted by the models trained with traditional and bespoke data augmentation, respectively. False-positive views are defined as in [1] as images that are labeled as NT, but are predicted by the model as one of the five views of interest (Fig 2b). Bespoke data augmentation resulted in a model that predicted about 20 percent fewer false-positive views than traditional data augmentation (626±11 images vs 779±50 images, p-value < 0.01).

## Discussion

In this work, we explore the utility of using domain knowledge in medical images to design bespoke data augmentations for neural network training. The fetal view classification use case provides a helpful demonstration of strategies and potential pitfalls in design, implementation, and benchmarking, due to the size and composition of the available training datasets and the statistical rigor provided in the experiments presented. Given the nature of fetal imaging, a cut-paste strategy was a reasonable bespoke data augmentation.

Training using offline-generated hybrid image data produced comparable test performance with only 13 percent of data used in the original model. Especially when the ability to apply traditional data augmentation may be limited, bespoke data augmentation has the potential to generate thousands of new labeled training images from a limited amount of labeled data. Furthermore, we then demonstrated feasibility in generating hybrid images online during model training, when previous cut-paste approaches to date were computationally prohibitive. In using online cut-paste generation, we further showed that the cut-paste technique alone provided sufficient data augmentation to provide regularization to model training. In a direct comparison between traditional and cut-paste data augmentation strategies, test performance was comparable overall, showing that when applied in an online manner, bespoke data augmentation is a valid and sufficient method for data augmentation. In the experiments shown, bespoke data augmentation resulted in fewer false-positive view predictions compared to traditional data augmentation. We hypothesize this is because blurring and shearing in traditional data augmentation has the potential to degrade important anatomic structures.

Efficient use of training data in model training can lighten data labeling burden, especially when combined with other strategies for training dataset curation [21]. This is particularly advantageous in the medical domain where there is a scarcity of data and experts to label this data.

Furthermore, bespoke data augmentation is a valid strategy to mitigate class imbalance in available training data. In the experiments above, the model trained on hybrid data showed improved performance for the 3VT class which had the least amount of training data.

Bespoke data augmentation can be implemented online during model training or offline before training. In an online manner, our chosen bespoke augmentation, cut-paste, provides both data augmentation and training regularization, avoiding overfitting even when no other data augmentation is used. Offline implementation provides more granular control over data sampling strategy and seeds can be used for the random combinations to ensure reproducibility. In this work, we expand experimental testing and statistical rigor for online bespoke data augmentation compared to prior work [9–16]. We also make available code for online bespoke data augmentation that can be adapted to the user’s augmentation of choice.

With respect to the particular bespoke augmentation chosen for this work, design of the cut-paste strategy comes with a caveat as it is heavily dependent on the effectiveness of the thorax detector. The performance of the thorax detector is not uniform across all the classes. For the NT class especially, there are many images which have no thorax in the frame. Applying quality control rules was generally effective in keeping the outliers from the thorax detector output but is not foolproof. The imbalance of the original training data combined with the class-specific performance of the thorax detector make this strategy suboptimal to use for the six-view classifier. However, the class imbalance also reflects the real-world class distribution of fetal ultrasound and echo images and we have tried to accommodate it as well as possible while maintaining experimental rigor. For future work, it would be best to consider domain-guided strategies that can be applied uniformly across all classes for optimal results.

Overall, we find that the ability to design and implement bespoke data augmentations for deep learning tasks in medical imaging expands the researcher’s toolbox for training models that are high-performance, clinically relevant, and data-efficient.

## Supporting information

S1 Table Example training batch to demonstrate sampling strategy for balancing original and augmented images at training time. (DOCX)

S1 Fig Loss plots for training with cut-paste and traditional data augmentation. Loss plots for training with balanced cut-paste (blue) and traditional (red) data augmentation. Training with traditional data augmentation overfits the training data. We performed three replicates for both (error in light blue and light red, respectively).
(DOCX)

S1 File (ZIP)
