# Supplementary material for: Domain-guided data augmentation for deep learning on medical imaging
Source: PLoS One. 2023 Mar 23;18(3):e0282532. doi: 10.1371/journal.pone.0282532 (PMC10035842; doi:10.1371/journal.pone.0282532)
Supplement: S1 Table — (DOCX) [file pone.0282532.s001.docx]

**Supporting information**

**S1 Table. Example training batch to demonstrate sampling strategy for balancing original and augmented images at training time.**

| View | Cut-paste eligible | Cut-paste data augmentation | Traditional data augmentation |
| --- | --- | --- | --- |
| 3VT | Yes | Cut-pasted hybrid & unchanged | Traditional DA & unchanged |
| 3VT | No | Unchanged only | Unchanged only |
| 3VV | Yes | Cut-pasted hybrid & unchanged | Traditional DA & unchanged |
| 3VV | Yes | Cut-pasted hybrid & unchanged | Traditional DA & unchanged |
| LVOT | Yes | Cut-pasted hybrid & unchanged | Traditional DA & unchanged |
| LVOT | Yes | Cut-pasted hybrid & unchanged | Traditional DA & unchanged |
| A4C | Yes | Cut-pasted hybrid & unchanged | Traditional DA & unchanged |
| A4C | Yes | Cut-pasted hybrid & unchanged | Traditional DA & unchanged |
| ABDO | No | Unchanged only | Unchanged only |
| ABDO | Yes | Cut-pasted hybrid & unchanged | Traditional DA & unchanged |
| NT | Yes | Cut-pasted hybrid only | Traditional DA only |
| NT | No | Unchanged only | Unchanged only |
| NT | Yes | Cut-pasted hybrid only | Traditional DA only |
| NT | No | Unchanged only | Unchanged only |

If a target view image is cut-paste eligible it is passed in both its hybrid and unchanged form. However, a cut-paste eligible NT image is only passed in its hybrid or Traditional DA form. DA, Data Augmentation; 3VT, three-vessel trachea; 3VV, three-vessel view; LVOT, left ventricular outflow tract; A4C, axial four-chamber; ABDO, abdomen; NT, non-target.
