# Supplementary material for: Domain-guided data augmentation for deep learning on medical imaging
Source: PLoS One. 2023 Mar 23;18(3):e0282532. doi: 10.1371/journal.pone.0282532 (PMC10035842; doi:10.1371/journal.pone.0282532)
Supplement: S1 Fig — Loss plots for training with balanced cut-paste (blue) and traditional (red) data augmentation. Training with traditional data augmentation overfits the training data. We performed three replicates for both (error in light blue and light red, respectively). (DOCX) [file pone.0282532.s002.docx]

**Supporting information**


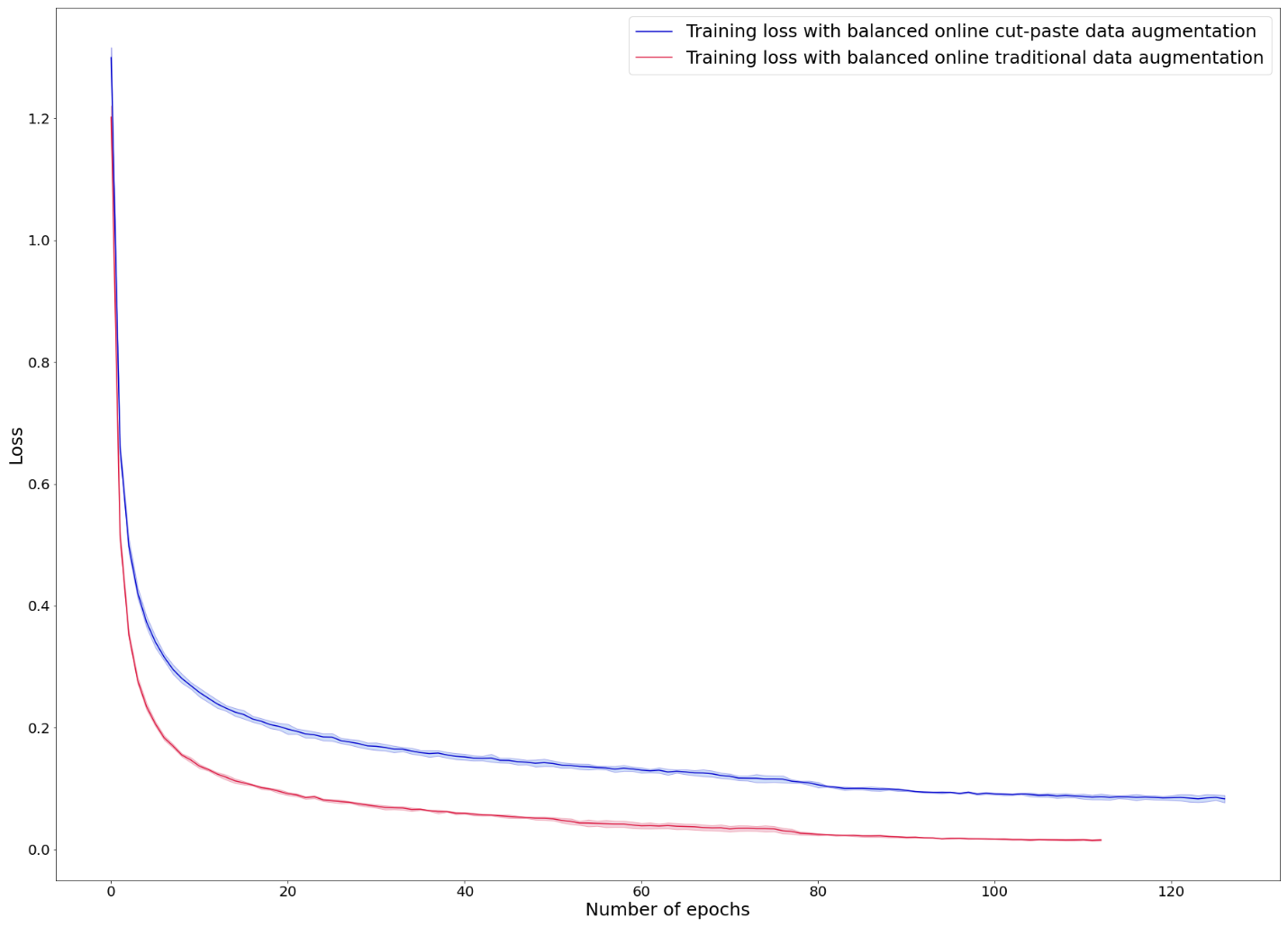


**S1 Fig. Loss plots for training with cut-paste and traditional data augmentation.** Loss plots for training with balanced cut-paste (blue) and traditional (red) data augmentation. Training with traditional data augmentation overfits the training data. Three replicates are shown for each experiment (error in light blue and light red).
