# Supplementary figures and images for: Domain-guided data augmentation for deep learning on medical imaging
Source: PLoS One. 2023 Mar 23;18(3):e0282532. doi: 10.1371/journal.pone.0282532 (PMC10035842; doi:10.1371/journal.pone.0282532)

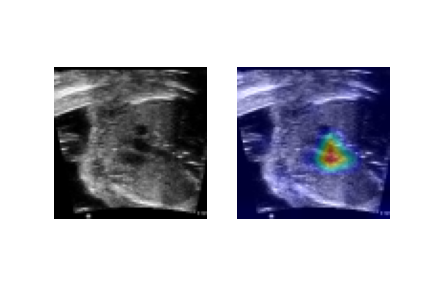

Supplement: S1 File — (ZIP) [file pone.0282532.s003.zip › Athalye_et_al_2023_figures_data/fig_1/example3_donor.png]

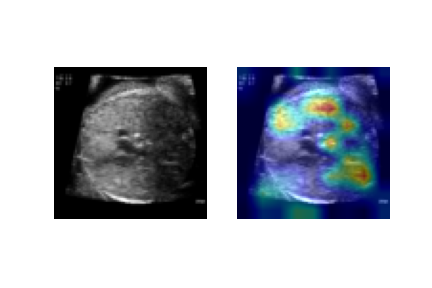

Supplement: S1 File — (ZIP) [file pone.0282532.s003.zip › Athalye_et_al_2023_figures_data/fig_1/figure1b_hybrid_example_4_gradcam.png]

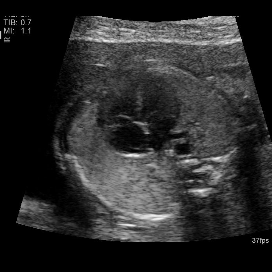

Supplement: S1 File — (ZIP) [file pone.0282532.s003.zip › Athalye_et_al_2023_figures_data/fig_1/figure1c_orig_example_A4C.png]

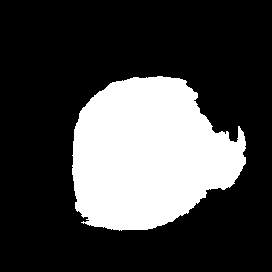

Supplement: S1 File — (ZIP) [file pone.0282532.s003.zip › Athalye_et_al_2023_figures_data/fig_1/figure1a_pred_mask.png]

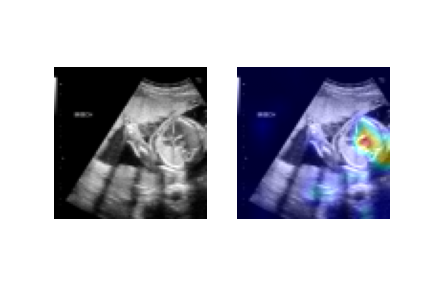

Supplement: S1 File — (ZIP) [file pone.0282532.s003.zip › Athalye_et_al_2023_figures_data/fig_1/figure1c_orig_example_A5C_gradcam.png]

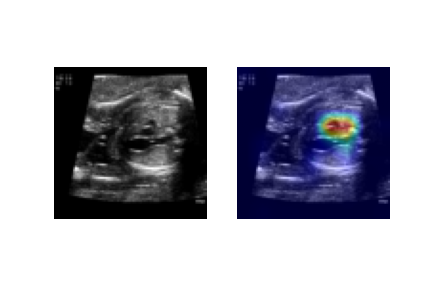

Supplement: S1 File — (ZIP) [file pone.0282532.s003.zip › Athalye_et_al_2023_figures_data/fig_1/figure1c_orig_example_3VV_gradcam.png]

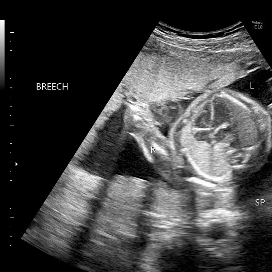

Supplement: S1 File — (ZIP) [file pone.0282532.s003.zip › Athalye_et_al_2023_figures_data/fig_1/figure1c_orig_example_A5C.png]

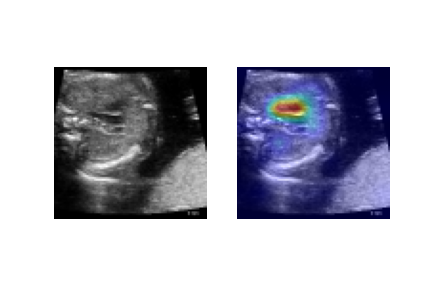

Supplement: S1 File — (ZIP) [file pone.0282532.s003.zip › Athalye_et_al_2023_figures_data/fig_1/example5_donor.png]

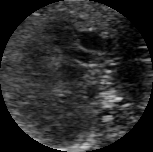

Supplement: S1 File — (ZIP) [file pone.0282532.s003.zip › Athalye_et_al_2023_figures_data/fig_1/figure1a_thorax_image.png]

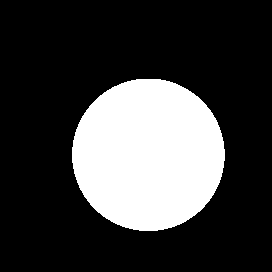

Supplement: S1 File — (ZIP) [file pone.0282532.s003.zip › Athalye_et_al_2023_figures_data/fig_1/figure1a_circle_mask.png]

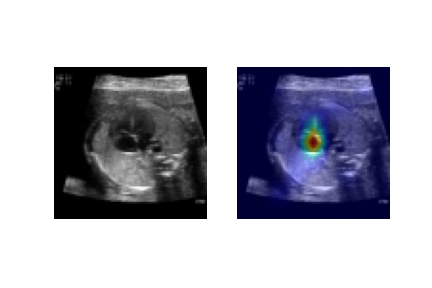

Supplement: S1 File — (ZIP) [file pone.0282532.s003.zip › Athalye_et_al_2023_figures_data/fig_1/figure1c_orig_example_A4C_gradcam.png]

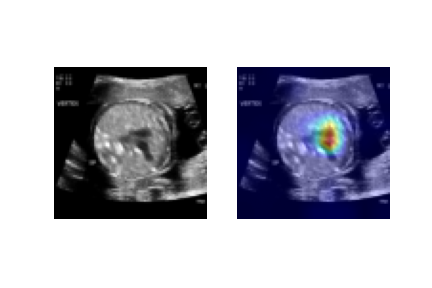

Supplement: S1 File — (ZIP) [file pone.0282532.s003.zip › Athalye_et_al_2023_figures_data/fig_1/figure1b_hybrid_example_1_gradcam.png]

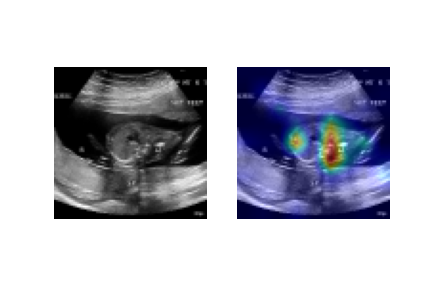

Supplement: S1 File — (ZIP) [file pone.0282532.s003.zip › Athalye_et_al_2023_figures_data/fig_1/example2_donor.png]

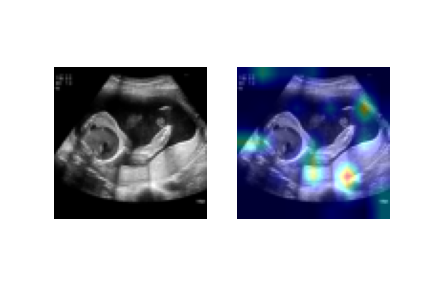

Supplement: S1 File — (ZIP) [file pone.0282532.s003.zip › Athalye_et_al_2023_figures_data/fig_1/figure1b_hybrid_example_2_gradcam.png]

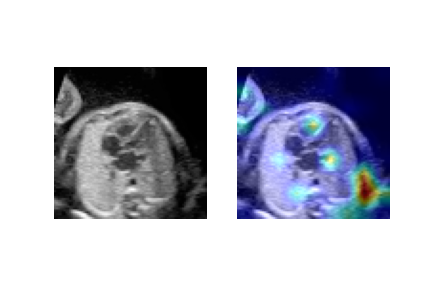

Supplement: S1 File — (ZIP) [file pone.0282532.s003.zip › Athalye_et_al_2023_figures_data/fig_1/example3_acceptor.png]

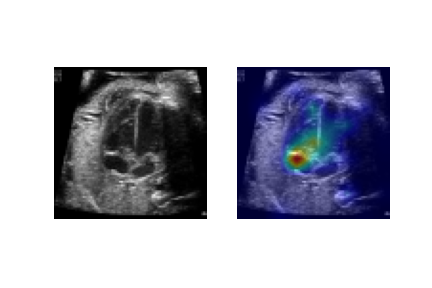

Supplement: S1 File — (ZIP) [file pone.0282532.s003.zip › Athalye_et_al_2023_figures_data/fig_1/example4_acceptor.png]

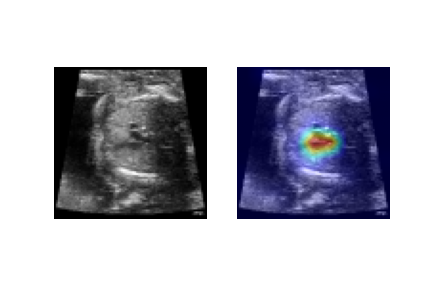

Supplement: S1 File — (ZIP) [file pone.0282532.s003.zip › Athalye_et_al_2023_figures_data/fig_1/example4_donor.png]

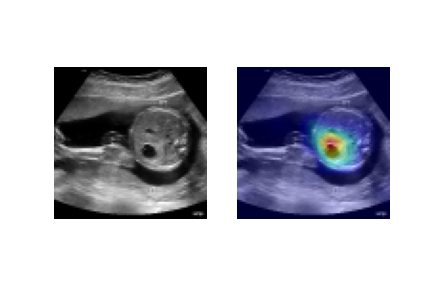

Supplement: S1 File — (ZIP) [file pone.0282532.s003.zip › Athalye_et_al_2023_figures_data/fig_1/example5_acceptor.png]

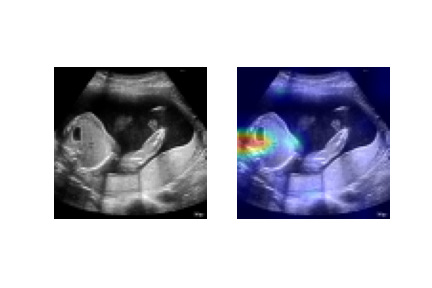

Supplement: S1 File — (ZIP) [file pone.0282532.s003.zip › Athalye_et_al_2023_figures_data/fig_1/example2_acceptor.png]

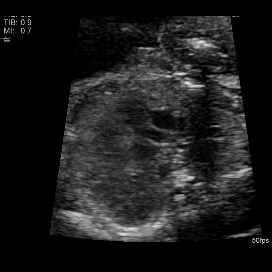

Supplement: S1 File — (ZIP) [file pone.0282532.s003.zip › Athalye_et_al_2023_figures_data/fig_1/figure1a_orig_image.png]

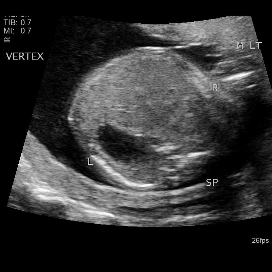

Supplement: S1 File — (ZIP) [file pone.0282532.s003.zip › Athalye_et_al_2023_figures_data/fig_1/figure1c_orig_example_ABDO.png]

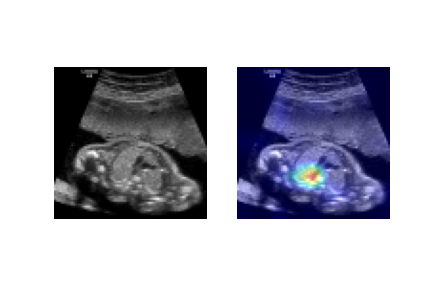

Supplement: S1 File — (ZIP) [file pone.0282532.s003.zip › Athalye_et_al_2023_figures_data/fig_1/example1_donor.png]

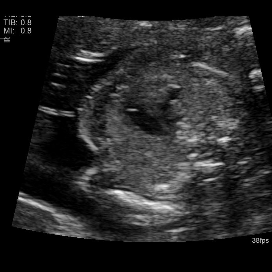

Supplement: S1 File — (ZIP) [file pone.0282532.s003.zip › Athalye_et_al_2023_figures_data/fig_1/figure1c_orig_example_3VT.png]

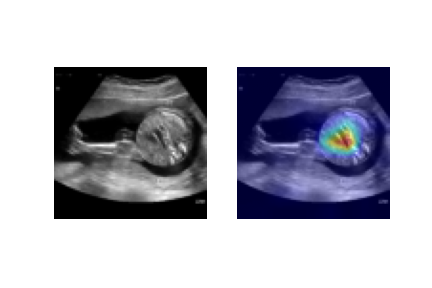

Supplement: S1 File — (ZIP) [file pone.0282532.s003.zip › Athalye_et_al_2023_figures_data/fig_1/figure1b_hybrid_example_5_gradcam.png]

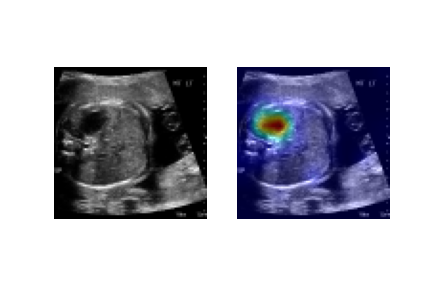

Supplement: S1 File — (ZIP) [file pone.0282532.s003.zip › Athalye_et_al_2023_figures_data/fig_1/example1_acceptor.png]

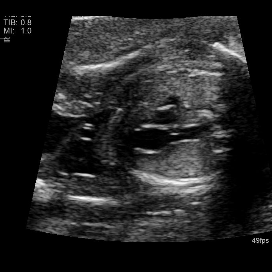

Supplement: S1 File — (ZIP) [file pone.0282532.s003.zip › Athalye_et_al_2023_figures_data/fig_1/figure1c_orig_example_3VV.png]

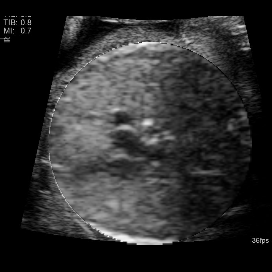

Supplement: S1 File — (ZIP) [file pone.0282532.s003.zip › Athalye_et_al_2023_figures_data/fig_1/figure1b_hybrid_example_4.png]

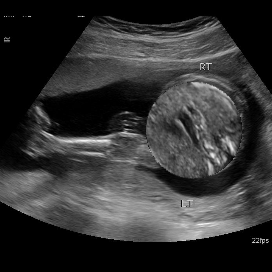

Supplement: S1 File — (ZIP) [file pone.0282532.s003.zip › Athalye_et_al_2023_figures_data/fig_1/figure1b_hybrid_example_5.png]

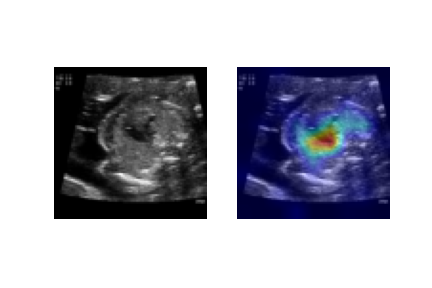

Supplement: S1 File — (ZIP) [file pone.0282532.s003.zip › Athalye_et_al_2023_figures_data/fig_1/figure1c_orig_example_3VT_gradcam.png]

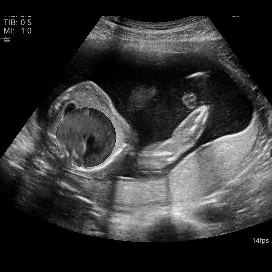

Supplement: S1 File — (ZIP) [file pone.0282532.s003.zip › Athalye_et_al_2023_figures_data/fig_1/figure1b_hybrid_example_2.png]

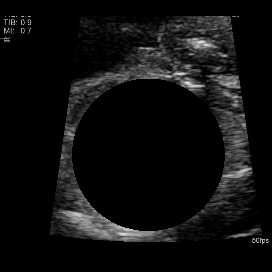

Supplement: S1 File — (ZIP) [file pone.0282532.s003.zip › Athalye_et_al_2023_figures_data/fig_1/figure1a_cavity_image.png]

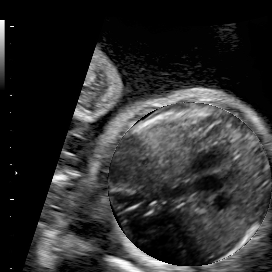

Supplement: S1 File — (ZIP) [file pone.0282532.s003.zip › Athalye_et_al_2023_figures_data/fig_1/figure1b_hybrid_example_3.png]

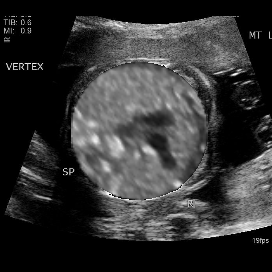

Supplement: S1 File — (ZIP) [file pone.0282532.s003.zip › Athalye_et_al_2023_figures_data/fig_1/figure1b_hybrid_example_1.png]

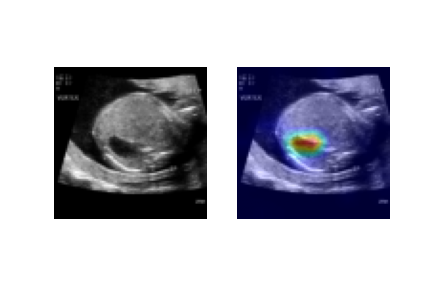

Supplement: S1 File — (ZIP) [file pone.0282532.s003.zip › Athalye_et_al_2023_figures_data/fig_1/figure1c_orig_example_ABDO_gradcam.png]

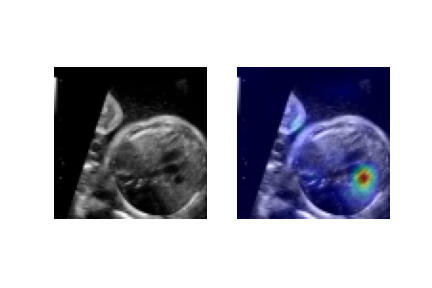

Supplement: S1 File — (ZIP) [file pone.0282532.s003.zip › Athalye_et_al_2023_figures_data/fig_1/figure1b_hybrid_example_3_gradcam.png]
